# Brain-Derived Neurotrophic Factor – The Protective Agent Against Neurological Disorders

**DOI:** 10.2174/1871527322666230607110617

**Published:** 2024-06-15

**Authors:** Prathyusha Koyya, Ram Kumar Manthari, Santhi Latha Pandrangi

**Affiliations:** 1 Department of Biotechnology, GITAM School of Science, Gandhi Institute of Technology and Management (Deemed to be University), Visakhapatnam-530045, Andhra Pradesh, India;; 2 Department of Biochemistry and Bioinformatics, GITAM School of Science, Gandhi Institute of Technology and Management (Deemed to be University), Visakhapatnam-530045, Andhra Pradesh, India

**Keywords:** Autophagy, cognitive impairment, brain-derived neurotrophic factors, BDNF receptors, neurogenesis, synaptic plasticity

## Abstract

The burden of neurological illnesses on global health is significant. Our perception of the molecular and biological mechanisms underlying intellectual processing and behavior has significantly advanced over the last few decades, laying the groundwork for potential therapies for various neurodegenerative diseases. A growing body of literature reveals that most neurodegenerative diseases could be due to the gradual failure of neurons in the brain's neocortex, hippocampus, and various subcortical areas. Research on various experimental models has uncovered several gene components to understand the pathogenesis of neurodegenerative disorders. One among them is the brain-derived neurotrophic factor (BDNF), which performs several vital functions, enhancing synaptic plasticity and assisting in the emergence of long-term thoughts. The pathophysiology of some neurodegenerative diseases, including Alzheimer’s, Parkinson’s, Schizophrenia, and Huntington’s, has been linked to BDNF. According to numerous research, high levels of BDNF are connected to a lower risk of developing a neurodegenerative disease. As a result, we want to concentrate on BDNF in this article and outline its protective role against neurological disorders.

## 1 INTRODUCTION

During the 1950s, studies determined that nerve growth factors (NGF) play a vital role in invigorating sensory and sympathetic nerve function by promoting viability, development, and stimulation. Subsequently, various members of the neurotrophin family, such as neurotrophin-3 (NT-3) and neurotrophin-4/5 (NT-4/5), have been identified with trophic effects on both the peripheral and central nervous systems [1]. During stressful circumstances such as brain ischemia, hypoglycemia, glutamatergic stimulation, and neurotoxicity [2], neurotrophins are neuroprotective [3]. They are essential regulators of neuronal survival, dendrite development [4], differentiation [5], and neuroplasticity [6]. Among the neurotrophins, brain-derived neurotrophic factor (BDNF) is known to activate and restrict neurogenesis or develop novel nerve cells from the nerve stem cells [2, 7]. In 1982, studies with the pig brain revealed that BDNF was synthesized by nerve cells in the dorsal root ganglion (DRG). The hippocampal and cerebral cortical in mature mammals have the maximum BDNF activity levels, broadly distributed throughout the brain [6], the olfactory bulb (OB), the basal forebrain [8, 9], the brainstem, the mesencephalon, the hypothalamus, and the spinal cord [2]. Low BDNF levels detected during early-stage in Parkinson's disease (PD) [10], Huntington's disease (HD) [11], Alzheimer’s Disease (AD), and multiple sclerosis (MS) [12] may be associated with underlying pathogenic mechanisms. Being neuroprotective, BDNF significantly regulates energy homeostasis, too [2].

## 2 STRUCTURE OF BDNF

Human NGF, NT-3, and NT-4/5 match roughly 50% of overall amino acid affinity with the polished version of the human’s BDNF, which is localized to chromosome 113. The pro-region of every neurotrophin encloses an N-linked glycosylation site, a proteolytic cleavage site, and a unique 3-dimensional configuration comprised of 2 groups of alternatively spliced β-strands. Thus, every neurotrophin is a homodimer linked to a single peptide after the start codon [1]. NT-3 is robustly expressed in the grey matter of motor neurons in the spinal cords. As a result, NT-3 plays a crucial role in these motor neurons’ survival and regular operation, especially in the ventral horn [13].

The pro-BDNF protein is encrypted by a 3' exon in the mouse and rat BDNF genes, along with eight 5' noncoding exons (exons I-VIII). One 5' exon controlled by distinct promoters is cleaved into the protein-coding exon for every BDNF transcript. Exon IXA, the 5' extended protein-coding exon, is the only exon expressed in the novel BDNF transcript of mice and rats. A new nomenclature scheme has been introduced for the BDNF exons in mice and rats. According to the old nomenclature, the previous exon III equates to exon IV; the former exon IV has now become exon VI, and the previous coding exon V has become exon IX [14]. It has been hypothesized that the rat BDNF gene undergoes cryptic splicing inside exon II to synthesize the genes IIA, IIB, and IIC [2]. As shown in Fig. (**1**) , human BDNF structural organization is highly identical to rat and mouse BDNF. Exons II and VII of the BDNF mRNA are primarily expressed in the brain; Exons IV and IX of BDNF transcripts are highly expressed in the human heart, placenta, and prostate, whereas the lungs, kidney, muscles, and stomach are primary sites for exon VI transcripts. Based on the in-situ hybridization studies, BDNF mRNA is strongly expressed in the brain. The activity of BDNF is minimal throughout embryonic development, significantly improves upon birth, and later declines in adulthood [2, 15].

## 3 MECHANISMS OF ACTION

### 3.1 BDNF Receptors

Every neurotrophin binds to one or several distinct Trk receptors, which have a strong affinity towards neurotrophins. For instance, NGF stimulates the TrkA receptor, NT-3 primarily stimulates the TrkC receptors, and BDNF, along with NT-4/5, activates the TrkB receptor. The TrkB receptor emerges in 2 isozymes: the standard-length receptor conjugated protein, *i.e*., gp145TrkB (145kDa), and the condensed version gp95TrkB (95 kDa) that deficits the tyrosine kinase domain and p75NTR commonly defined as LNGFR (low-Affinity Nerve Growth Factor Receptor [2, 16]. Both pro- and anti-trophic activities, which include the initiation of apoptosis in developing nerve cells, rely on the p75NTR [17]. The brain has high levels of BDNF and gp145TrkB, which are its receptors [2].

### 3.2 Activation of TrkB

The neurotrophic factors also synchronize cell fate determination, axonal and dendritic development, pruning, and the synthesis of proteins involved in neurogenesis, including ionotropic receptors, neuropeptide transmitters, and transmitter anabolic enzymes [18]. In humans, neurotrophic tyrosine kinase is expressed by the NTRK2 gene. The matured protein upshots TRKA, TRKB, and TRKC are produced in response to post-translational glycosylation of the extracellular domains of the TRK superfamily receptors, which are generated as progenitor proteins. G protein, including Ras and MAPK, when initiated, modulates the PI3K and phospholipase-C-γ (PLC-γ) cascades. In the spinal cord, signal initiation occurs more quickly than inactivation; initiation takes place in 120 seconds, while inactivation takes place in about half an hour [19, 20]. The production of intermediates in these signal transductions contributes to constraining the Trk receptor-mediated signaling, and membrane mobility control the positioning of various signaling component [21].

### 3.3 The Significance of BDNF in Signal Transduction Cascades in a Mechanistic View

**Fig. (2):**
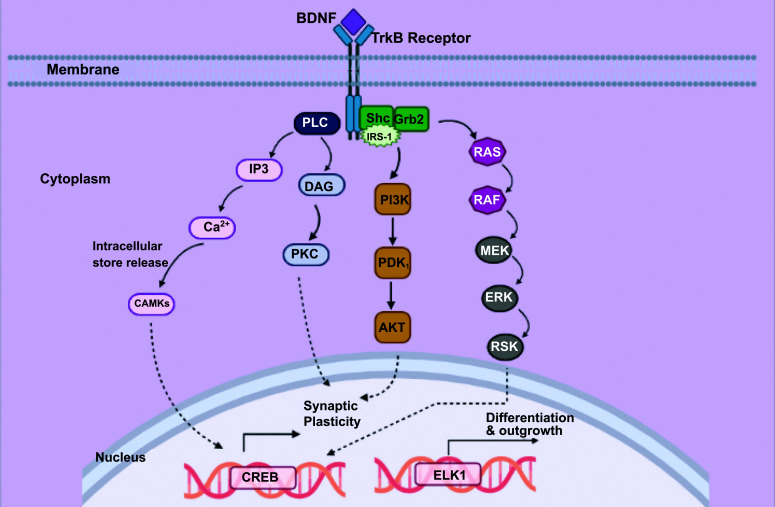
Activation of signaling pathways upon BDNF binding to the TrkB receptor. Tyrosine kinase B (TrkB) is recognized as the high-affinity receptor for BDNF. Upon binding, BDNF triggers the mobilization of proteins, leading to the activation of three distinct signaling pathways.

Tyrosine kinase B is regarded as the BDNF’s high-affinity receptor. Proteins are mobilized when BDNF binds to TrkB, primarily activating three distinct signaling pathways (Fig. **2**). In the first cascade, the growth factor receptor-bound protein 2 (grb2) interacts with Ras-containing GTPase activity to activate extracellular signal-regulated kinase (ERK), which leads to the activation of the Shc adaptor protein. The CREB transcription element is triggered by active ERK to stimulate the mitogen-activated protein kinase MAPK/ERK/RSK pathway. In the second cascade, insulin receptor substrate-1 (IRS-1), phosphatidylinositol 3-kinase (PI3K), pyruvate dehydrogenase kinase-1 (PDK-1), and protein kinase B (Akt) are sequentially activated. The phospholipase C (PLC) cascade is involved in the third cascade, which leads to the generation of inositol-1,4,5-triphosphate (IP3) and diacylglycerol (DAG). Ca^2+^/CaMK II is regulated *via* the PLC/IP3 cascade, which also induces calcium to be generated from intracellular deposits. In contrast, DAG induces synaptic plasticity by stimulating PKC. The activation of genes encoding proteins engaged in nerve survival, plasticity, differentiation, stress tolerance, and cell existence is monitored by transcriptional regulators as cAMP-response-element-binding protein (CREB) and CREB-binding protein (CBP), which are induced by BDNF signaling cascades.

#### 3.3.1 RAS/MAPK/ERK Pathways

Followed by the dimerization of the TrkB receptor and interaction with the BDNF, tyrosine residues undergo phosphorylation. Then, the Shc adaptor protein and Phospholipase C (PLC) plot for the src homology domain [19]. SOS docks Shc with the receptor and its adaptor protein Grb2 leading to RAS activation [2]. Activated RAS triggers the RAS/MAPK-ERK pathway, PI3K pathway, and PLC pathway [22]. By activating several genes responsible for neuronal vitality and obstructing programmed cell death, MAPK/ERK plays a critical role in the emergence and viability of nerves [19]. In undeveloped neural cells, it has been proven that BDNF and indigenous Ras protein activity can suppress MK801-stimulate neural damage [23].

#### 3.3.2 IRS-1/PI3K/AKT Pathways

Insulin receptor mobilization, substrate-1 (IRS-1/2), PI3K, and protein kinase B (Akt) are alternative mechanisms that BDNF triggers [19]. When the regulatory subunit of PI3K binds to the receptors or receptor-binding proteins on the membrane, its catalytic subunit activates, followed by the recruitment of PDK1 and AKT by activated PI3K [24]. Recruited AKT plays a major role in synaptic plasticity maintenance in the healthy nervous system [25]. Besides, it is well-reported that Ras halts cell death and promotes cell differentiation [19]. Thus, intracellular coupling of Ras with PI3K-Akt *i.e*., Ras-PI3K-Akt cascade, enables sympathetic nerves to survive [26]. RAS-PI3K-Akt cascade inhibition at any stage significantly decreases sympathetic nerve cells’ ability to withstand NGF, indicating that PI3K pathways activated by BDNF contribute a crucial function in the activation of pro-survival genes essential for cell culture [2].

#### 3.3.3 PLC/DAG/IP3 Pathways

Interaction of BDNF with the Trk receptor results in the phosphorylation of the adaptor protein PLC-gamma. This causes the digestion of membrane lipids into inositol 1,4,5 triphosphate (IP3), which improves a significant rise in intracellular calcium concentration and diacylglycerol (DAG) [2]. Protein kinase C, essential for the MAPK/ERK signaling cascade associated with neurite outgrowth, is regulated by DAG in turn [27-29].

## 4 NEUROTROPHIC FACTORS (NTFS)

Small proteins called NTFs can have the ability to therapy human neurodegenerative disorders [30]. In the last 25 years, clinical studies in neurodegenerative disorders have demonstrated the therapeutic potential of neurotrophic factors [31]. Specific neuronal populations are controlled and assisted in their classification, progression, and viability by neurotrophic factors, which are secreted proteins [32]. The two obscure NTF families in the transforming growth factor-β (TGF-β) superfamily of growth factors, glial cell line-derived neurotrophic factor (GDNF) and neurturin (NRTN), have been demonstrated in the animal models of PD [33]. GDNF, which binds to GFR1, and NRTN, which binds to GFR2, enhance DA survival while having a wide range of effects on various populations of neurons in the CNS and PNS. Most interestingly, GDNF and NRTN play crucial regulatory roles in the development of parasympathetic neurons and are necessary for developing the enteric nervous system. Most recently, Barker *et al*. (2020) reported that therapy with GDNF failed to attain its chief endpoint in placebo-regulated trials [34]. In people with Parkinson’s disease (PD), neurotrophic factors could improve stimulation and promote the longevity of cells by enhancing the dopamine system’s activity [31]. Gene therapy is used to enhance the therapeutic efficiency of NTFs. Gene therapy has two main forms, including *in vivo* and *ex vivo*. *Ex vivo* studies have relied on bioengineered culture cells to develop curative proteins known as NTFs. On the contrary, *in vivo*, gene therapy was considered when patients receive direct gene transfer of a gene into their endogenous cells [30].

Based on recent studies, NTFs, however, hold significant therapeutic promise. In addition to replenishing the target neurons, NTFs can produce neurotrophins to enhance the environment for the regeneration of nerves and restoration [35]. NTFs have a wide range of impacts on neuronal activity, including synaptic plasticity & cognition, which imply potential therapeutic efficacy in neurodegenerative conditions linked to CI, for instance, AD [36]. The loss of specific neuron types does occur in various neurodegenerative disorders, including dopaminergic neurons in PD, projection nerve cells in HD, cholinergic nerve cells in AD, and motor cells in ALS. Therefore, the use of cell-specific NTFs in therapeutic activities should be prioritized in further studies, as this innovative therapy approach is anticipated to benefit patients with neurodegenerative disorders [35].

## 5 BMP- THE REGULATOR OF BDNF

BMPs not only modulate the generation, segmentation, and survival of cells but also synchronize their interactions to attain the desired reaction [37, 38]. BMPs are isolated from a demineralized bone intercellular substrate to determine their proficiency to persuade in the emergence of young bone *in vivo*. This unveiled that bone replacement of cartilaginous tissue and the unique advancing of signaling pathways (Fgf, Wnt, and BMP) are vital for perceiving skeletal genesis [39, 40]. Monocytes, epithelial cells, mesenchymal cells, and nerve cells are among the diverse cell kinds involved in various biotic procedures that are presumed to incorporate BMPs.

### 5.1 BMP-smad Signaling

To stimulate the smad signaling cascade, BMPs impose their cellular reaction through heteromeric compounds of particular BMP type I and type II serine/threonine kinase receptors, for instance, BMPRIA and BMPRII [41, 42]. Activin receptor-analog kinase (ALK1-7), often known as one of the seven type I and five type II receptors that, has been distinguished so far [43]. BMP signaling can also occur through a non-canonical pathway, *i.e*., the non-Smad mechanism which acts by ligand-engaged receptors. These non-Smad pathways comprise GTPase signaling, MAPK, and phosphatidylinositol-3-kinase/AKT [44].

Three functional groups are invaded by eight unique Smad proteins. The receptor-regulated Smads, also known as (R Smads) 1, 2, 3, 5, and 8, comprise the first group. The only Smad in the second functional group, called the common mediator Smads (co-Smads) is pSmad4, and the inhibitory Smads, also known as (I-Smads), 6 and 7, make up the third functional group [45]. As shown in Fig. (**3**) , when a ligand binds to the heterotetrameric receptor complex, type II receptors phosphorylate the type I receptors at serine and threonine residues [37]. A heterodimeric compound is formed due to the binding of co-Smad 4 and phosphorylated R-Smads [46]. The R-Smads and Co-Smad 4 complex translocates to the nucleus, binds to DNA, and regulates the transcription of target genes to conclude the signaling cascade [37]. However, inhibitory Smads (I-smads) antagonistically mediate the activities of R-Smads and co-Smad-4 [47]. I-smads show their activity by triggering the proteasomal degradation of Smad 1/5, type I TGF-β receptors (TβRI), and also type I BMP receptors (BMPRI) [48]. A cytosolic loop defined as the L45 loop, which is situated next to the serine/glycine motif on type I receptors and is involved in R-Smad joining, has also been spotlighted in various research on the structural and functional aspects of the BMPRs [49].

Receptors bind distinct ligands from single or more TGF-β subfamilies; however, a specific TGF-β ligand can also bind to various TGF-β receptors of furthermore subtypes. This phenomenon is termed “ligand-to-receptor” promiscuity [50]. Moreover, various ligand types require the interaction between type-I and type-II receptors [37]. Two well-known type-I BMP receptors (BMPRs) have been reported. They are BMPR1A (also known as ALK3), a serine-threonine kinase type I receptor, and BMPR1B (defined as ALK6) [51]. The type-II BMP receptors are the activin type II receptors A (ActRIIa/ACVR2a) and B (ActRIIb/ACVR2b) as well as the type-II BMP receptor (BMPR2) [52]. The TGF-β type 1 (ALK 5) and activin type I receptors phosphorylate smads 2 and 3; several BMP/GDF receptors phosphorylate smads 1, 5, and 8 [53].

Novel studies have spotted that the BMPRs at the cell membrane's exterior are crucially regulated by dynamin-dependent endocytosis, which is key for the vitalization, temporal dynamics, and extent of the BMP signaling cascade [54]. Through phosphorylation, the type II receptors activate the type I receptors and frame a heterotetrameric compound comprised of 2 TGF-βRIs and 2 TGF-βRII [2]. The affinities of different BMP ligands for the receptors vary. For instance, BMP2 has more inflated affinity than BMPR2 during its binding to BMPR1A and BMPR1B. Likewise, GDF5 has a high affinity for BMPR2 and BMPR1B than BMPR1A. The primary concern of BMPR1B for GDF5 signaling is manifested by the fact that the GDF5 analogue for BMPR1B is 15 times greater than it is for BMPRIA [37, 55].

### 5.2 BMP Expression in the Nervous System

The temporal expression of BMPs and their receptors in emerging neurology has been distinguished by various investigations [37]. In the striatum, cerebral mantle, hippocampal, and substantia nigra (SN) of the brain, BMPR1 and BMPR2 are rigidly evident throughout middle age [56]. The mice ventral mesencephalon (VM) has disclosed the presence of BMPR1B and BMPR2 receptors since embryonic time (E) 14 forth, and this process has continued at leastwise post-natal day (PND) 90 [57]. From embryonic day 12, BMPR1B and BMPR2 transcripts are synthesized in the mice’s ventral mesencephalon [56]. It remarkably resembles BMPR temporal expression pattern and mimics the development of dopaminergic projections in the nigrostriatum. It has been reported that specific BMPs can mimic with NTFs mDA nerve cells in function. Goulding *et al*. (2019; 2020) reported that BMP2 is depicted in the human substantia nigra and can prevent the degeneration caused by α-synuclein and neurotoxins [37, 58].

## 6 BDNF REGULATES AUTOPHAGY

**Fig. (4):**
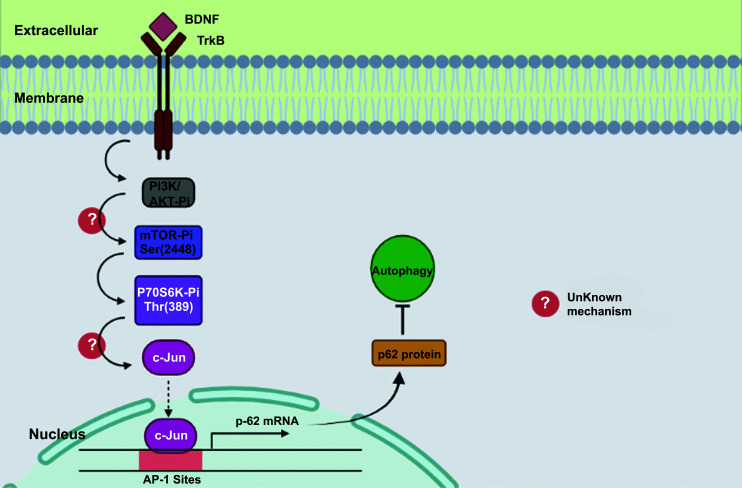
The stimulation of the TrkB signaling cascade appears to be the technique through which BDNF subdues autophagy.

Autophagy is decisive for neuronal integrity. Intense neuro deterioration and configurational fault in the pre and postsynaptic framework result from the drop of vital autophagic elements [59]. With concern to nutrition deficiency for the atrophy of supermolecules, autophagy is persuaded by the lysosomal mechanism to pioneer cellular endurance by suppressing the cells of blemished microorganisms and/or lethal microbes [60]. A chief regulatory mechanism for autophagy is mediated by the mammalian target of rapamycin (mTOR) [61]. In nutrient-rich environments, mTOR is zestful and boosts protein fusion to satisfy cellular anabolic urges. To impede broad protein fusion and promote autophagy, in exhausting growth circumstances, mTOR is immobilized [62]. Selective autophagy, which includes ubiquitin-binding proteins like p62/sequestome-1 (p62/SQSTM1), is a specific form of autophagic degradation [63]. According to various studies, p62 serves as specific autophagy by arranging vast inclusions/aggregates termed p62 bodies or sequestosomes. These inclusions/aggregates conscript and transfer ubiquitinated contents to the autophagosome for atrophy through an alliance between p62 and the autophagosomal membrane's inherent LC3-I [64]. Relying on the enhanced levels of beclin 1 and LC3-II, neurochemical studies in the rat striatal subjected to 3-NP through stereotaxic administration demonstrate mobilization of autophagy according to the *in vivo* criteria [59]. To ensure the BDNF-liaised neurorestorative outcomes versus mitochondrial malfunction, blocking of autophagy shift is indeed critical. The stimulation of the TrkB signaling cascade appears to be the technique through which BDNF subdues autophagy (Fig. **4**) [65].

mTOR/c-Jun-dependent initiation of p62 synthesis is leastways in the role by which BDNF constrains autophagy, providing neuroprotection against the mitochondrial constriction. A review by Chen *et al*. (2017) hypothesized that BDNF incites the synthesis of p62 in primary cortex nerve cells to subdue autophagy and strive for the neuroprotective benefit [60]. The enzyme kinase activates BDNF-incited c-Jun in cortical nerve cells [66]. The C-jun dropdown diminished BDNF-dependent p62 upregulation; moreover, BDNF provoked the phosphorylation of mTOR and the downstream regulator p70S6K [60]. In supplement to subduing p62 initiation, the mTOR inhibition by rapamycin slightly conceals BDNF-actuated c-Jun expression [67]. Du *et al*., in 2020, by employing mTOR/c-Jun reliant initiation of p62 synthesis, stated that BDNF impedes 3-NP-effected autophagy [68].

## 7 FUNCTIONS OF BDNF

### 7.1 Neurogenesis

Neurogenesis, the process by which new neurons are synthesized, is pivotal during embryo development, which also maintains in certain brain areas after birth and throughout our lifespan. BDNF’s gravity in the viability of the majority of peripheral sensory nerve cells, which also impacts the survival of a minor fraction of motor nerve cells throughout the development, is among the prime *in vivo* roles of BDNF discovered [69]. The neurotrophin BDNF promotes neural proliferation and governs a specific pattern of dendrite development. According to recent studies by Zagrebelsky *et al*. (2020), pyramidal nerve cells of the visual cortex and the hippocampus exhibit dendrite spines that are denser and more complex after long-term exogenous BDNF treatment *in vitro* [70]. Akt, the protein kinase B explored in AKT virus, the Ras-mitogen-activated protein kinase (MAPK), the cAMP/protein kinase A (PKA), and phosphatidylinositol-3-OH kinase (PI3K) cascade are all components of signaling pathways that BDNF triggers. Based on the kind of neural cell and the viability factors, these cascades contribute to cellular existence under factual circumstances [71]. The neural apoptosis triggered by caspase-3's activity and the resultant disintegration is restrained by BDNF. The continual BDNF administration for two weeks increased neurogenesis throughout the thalamus [2, 72], revealing the important role played by BDNF during neurogenesis. Also, dietary restriction improved the initial development of dentate granule nerve cells and enhanced neurogenesis in the dentate gyrus of mature rats [73].

### 7.2 Synaptic Plasticity

As the basis for memory, synapse, synaptic plasticity, efficacy, and neurological communication, BDNF performs a critical role in cognitive retention. Lack of BDNF signaling induces spine shortening in the striate body [19]. Through both pre-and post-synaptic processes, BDNF enhances excitable synaptic connections [74]. Pre-synaptic channel proceeding, which relies on NMDA (N-methyl D-aspartate) receptor stimulation, requisites BDNF [2]. Bathina *et al*. (2015) reported reduced long-term potentiation (LTP) upon decreased TrkB and BDNF secretions. Thus, it is hypothesized that BDNF facilitates the influx of Ca^2+^, that in order gives rise to post-synaptic BDNF, which in turn increases pre-synaptic vesicle cycling, therefore, improves LTP and neuroplasticity [2].

### 7.3 BDNF in Neurological Disorders

As discussed above, the fact that BDNF can increase neurogenesis and neuroplasticity suggests that it may play a considerable role in various neurological disorders [2]. A variety of neurological conditions known as neuron deterioration diseases, including Alzheimer's disease (AD), Parkinson's disease (PD), Huntington's disease (HD), and amyotrophic lateral sclerosis (ALS), are defined by the decline and loss of precise neuronal nuclei in the spine or cerebrum [75]. The fact that the pancreas, liver, and brain tissues all produce BDNF raises the possibility that type 2 diabetes Mellitus cognitive dysfunction, altered consciousness, and acquiring knowledge deficits are brought on by a BDNF shortage [76]. Studies with T2DM (Type 2 diabetes mellitus) individuals lacking depression revealed a significant decrease in BDNF concentrations, indicating that BDNF may be connected to depression in T2DM patients [77].

#### 7.3.1 BDNF in Schizophrenia

The fact that BDNF levels declined in long-term schizophrenia individuals affirms the idea that BDNF may have a role in the pathogenesis of the disease. Deficiency of this neurotrophic factor could lead to morphological and chemical disturbances in the brain, which would be the basis of the mental illness of neuropathology in schizophrenia, as supported by the reduced BDNF levels in schizophrenia patients. Excessive plasma BDNF concentrations are linked to betterment intellectual performance in schizophrenia individuals [78]. In addition, BDNF enhanced substantia nigra neural growth by 25% and suppressed the depletion of nigrostriatal nerve cells driven by the impairment at both caudate-putamen and paleostriatum induced by ibotenate for 21 days [2].

#### 7.3.2 BDNF in Alzheimer’s Disease (AD)

There is emerging proof that there is an association between reduced BDNF expression and AD occurrence. The assemblage of β-amyloid peptides (Aβ) and enhanced concentrations of hyperphosphorylated, cleaved tau neurotubules in the brain are considered as the chronic features of AD [19]. The emergence of neuritic plaques (NP) and neurofibrillary tangles (NFTs) appear as a result of divergent Tau protein phosphorylation and is characterized by mutated amyloid-β precursor protein (APP) metabolism. Neurodeterioration and the diagnostic manifestations of mental illness are caused by these occurrences [79]. Various research groups have demonstrated the significance of BDNF/TrkB transmission in amyloid processing. LTP and dendrite progression, which seem crucial for cognition, rely on BDNF. BDNF regulates LTP and LTD, which assists in synapse viability. The NMDA and AMPA glutamate receptors are altered to carry out this mechanism. In cultured neural cells, BDNF can lower the formation of amyloid-β, but it elevates when BDNF is deficient [19, 80]. Studies on animals have revealed that rats with Alzheimer’s seemed to have significantly diminished TrkB receptors throughout their cerebral mantle, which worsens their spatial cognition. Additionally, overexpression of shortened TrkB receptors prevented BDNF/TrkB signaling in rats with Alzheimer’s [19, 81]. Studies proved that invalidating NP and NFT generation may be possible by improving BDNF/TrkB signaling deficiencies in the cortical and hippocampal [82].

#### 7.3.3 BDNF in Huntington’s Disease (HD)

The genetically inherited HD, brought on by an increase in the frequency of CAG triplet codons in the HTT gene, which inscribes huntingtin, implies a function for BDNF in neurodegenerative pathologies. The intermediate spiny nerves of the striatum and the cerebral cortex are adversely impacted as a sign of this hereditary fault [83]. Apoptosome complex genesis and activity are critical for adequate CNS cell existence, where the HTT gene performs an essential role in both. In parallel, HD experiences a possible decrease in neuroprotective HTT activity, which leads to the proliferated intrusion of CNS neurons [84]. Regarding the striatal nerve cells that cease in HD, BDNF plays a pivotal role as an endurance element [85]. The synthesis of BDNF is affected by wild-type Huntington in normal and Huntington disease individuals [86]. The death rate of cultured neurons transfected with mutant HTT dwindled with BDNF treatment [87]. In crossbreeding HTT-mutant mice, BDNF heterozygous knockout led to the prior chronic outbreak and higher acute neuromuscular retardation [88]. Administration of BDNF upgraded muscle movements and inflated the viability of enkephalin-immunoreactive striatal neuronal cells in HTT-mutant rats [89]. Ampakines augmented BDNF concentrations in the hippocampal in HTT-mutant rats sustained LTP, and upgraded reminiscent, revealing the protective role of BDNF against HD [90].

#### 7.3.4 BDNF in Parkinson’s Disease (PD)

PD is a neurodegenerative condition that appears both in motor and non-motor manifestations. Dopaminergic nerve cells in the substantia nigra (SN) are degenerated, which is the central cause of the disease [91]. Parkinson’s disease is triggered by the breakdown of GABAergic nerve cells in the substantia nigra, which may also occur in Huntington’s disease [92, 93]. It is widely recognized that BDNF shields cultured GAB nerve cells from being lethal by neurotoxicity [2]. When individuals experience melancholia or PD, a decline in BDNF concentration in both hemoglobin and the nervous system is reported. A decline in BDNF levels within the plasma and cortex is followed by a rise of dopaminergic neuronal degradation in Parkinson’s disease, resulting in behavioral abnormalities, intellectual deficiencies, and psychiatric illness, which impairs memory [94]. According to findings on experimental PD animals, administration of BDNF may revive the impairment of dopaminergic nerve cells and D3 dopamine receptors. The nerve cells and the D3 receptor may not get depleted of BDNF in the mutant animal [19]. In primary hippocampal cultures, BDNF persuades signaling cascades that mediate the nuclear translocation of the transcription factor Nrf2, which shields cells from oxidative stress carried on by damage and inflammatory processes. The RAS-MAPK and PI3K-AKT signaling pathways regulated the defensive response [95]. By activating glycogen synthase kinase 3-β (GSK 3), AKT, and restraining CCND1, endoplasmic reticulum (ER) stress may induce nerve cells to die and lead to Parkinson’s disease [96, 97]. TrkB upregulation halted the processes by stimulating the AKT signaling cascade, upregulating CCND1, boosting phosphorylated GSK3, and thereby obstructing neuronal cell death [19]. Synuclein (α-syn) might impinge on BDNF/ TrkB signaling [98]. In addition, a decrease in the synthesis of TrkB and BDNF is also coupled with pathogenic α-syn alterations. It was discovered that the generation and proliferation of dendrite fibers in cortical neuronal cells depend on the inverted axon movement of BDNF/TrkB signaling endocytic vesicles. Furthermore, α-syn can decrease TrkB and persuading to become ubiquitinate [99]. However, the existence of BDNF might obstruct the association, limiting its loss [98]. A recent study by Ibrahim and his group (2022) has suggested the role performed by BDNF during PD treatment and diagnosis [19].

### 7.4 The Role of BDNF in Cognitive Impairment (CI)

Numerous reductions in the neuroendocrine, psychological, and autoimmune processes occur as a result of getting older. The maturity phase and its results are greatly influenced by various aspects, including genetic, epigenetic, and environmental elements [19]. An increased body of literature on cognitive impairment reveals that the hippocampus is crucial to cortical function, which might be related to changes in the composition and their consequent effects on the aged population’s variations in memory recall. Memory adherence is a concern of aging, and it's possible that aging may also influence choice-making [100]. Not always typically associated with the loss of neurons, changes in learning and memory abnormalities have also been linked to reduced BDNF synthesis and interaction [101]. Changes in the TrkB receptor result in impaired BDNF-induced LTP [102]. Ampakine infusion can lead to the synthesis of BDNF, which could regress alteration in brain plasticity [19]. Enhancing cognitive functions has been demonstrated by modulating AMPA receptor function by ampakines, which are implemented in reimposing LTP on the basal dendrons [103, 104]. TrkB and BDNF mRNA expression has been shown in numerous researches on both people and organisms to significantly drop in the aging brain [105, 106], revealing that decreased levels of BDNF and TrkB may lead to early aging. BDNF impacts cognition by influencing the amygdala hippocampal region that regulates response and cognition [107]. The relationship between BDNF, activity, and intellectual functioning, according to Wang *et al*., might have significant diagnostic inferences for halting and ameliorating dementia and memory dysfunction [108].

## 8 POTENTIAL IMPACT OF BDNF MARKERS

In the region-bound prospective research with 2131 individuals and a 10-year checkout, Hashimoto (2014) investigated the connection between the plasma levels of BDNF and the threat of experiencing mental deterioration. They observed that those with extremely high serum BDNF values are least prone to develop Alzheimer’s and dementia [109]. It’s fascinating to note that only women, applicants above the period of 80, and ones with academic backgrounds showed a significant correlation between plasma BDNF and the risk of Alzheimer’s and intrinsic dementia [19]. Due to their lower plasma concentrations of BDNF, which particularly contributes to AD growth, aged females had the greatest threat for developing Alzheimer’s, according to advanced investigations. Besides, preventative medications were employed to cure insanity, primarily the 7,8-dihydroxyflavone (7,8 DHF), a potent TrkB activator. Therapeutic applications aim to amplify low levels of BDNF in the plasma from normal people and those who are eventually prone to develop dementia or Alzheimer’s [110]. Apart from dementia and AD, the salient components influencing the transmission of BDNF concentrations within the system are nutritional restrictions and other bodily moments [111]. BDNF exploits the discerned connections between the risk of insanity and lifestyle as an intervention [112].

## 9 CLINICAL SIGNIFICANCE OF BDNF

From the above discussion, it is apparent that BDNF has various essential functions that may have substantial therapeutic relevance. Multiple neurodegenerative conditions, including Alzheimer's disease, Parkinson's disease, Huntington's disease, schizophrenia, and manic-depressive illness, are linked to a drop in BDNF activity [2]. Regular physical exercise enhances the BDNF signaling cascade, the inherent technique for boosting or limiting mental performance [113]. Manic depression has a successful therapy in lithium, which might have been shown to increase BDNF [114]. Unexpectedly, excessive exposure to BDNF throughout the hippocampal has been seen to fallout impulsive convulsions, eventually evolving into temporal zone epilepsy [2]. The longitudinal sections of intestinal smooth muscle cells express and secrete BDNF in their basal region. Pandit *et al*. (2020) reported that BDNF has a major involvement in the PLC cascades’ activation and regulation of the digestive tract function, revealing the tonic activity in disorders like dyspepsia and irritable bowel syndrome [22]. Autism spectrum disorder (ASD) was noticed with an adverse drop in the plasm BDNF level. Thus, BDNF expression in plasma can be a biomarker to detect autism in its initial phase [2].

## 10 NOVEL ADVANCEMENTS AND CHALLENGES

The potential of BDNF to access the cerebrospinal fluid with appealed concentration and prompt a healing reflex considers an essential therapeutic approach for neurodegenerative diseases. Though BDNF is a polar molecule with an adequate form, when injected auxiliary, it cannot penetrate the BBB [19, 115]. Consequently, BDNF must be administered directly into the brain tissue [116]. Administering BDNF into the brain’s ventricles or intrathecally into the CSF fails to probe into the brain parenchyma abundantly [117]. Nevertheless, when the BDNF is synthesized and unveiled in rats, the intensity of BDNF is surpassed [118]. However, this may not supply an adequate amount for an appropriate diagnosis in people. Consequently, the functioning of BDNF is equated along with the adverse effects, like comprising dysesthesia, caloric restriction, and Schwann cell migration through the superficial parenchymal area; the above adverse effects are results of BDNF’s diffusion to the peripheral region [19]. BDNF should be dispensed in an approach that could attain an adequate treatment in degenerated neurons with a constrained dispensation of other neurons to dwindle the side effects [119]. Additionally, the concentration should be sustained for an adequate period [120].

Diverse techniques are in practice to enable BDNF as a therapeutic agent, like the intracerebral dispensation of BDNF molecule [19, 121]. Various implanted instruments are used to inundate BDNF, with maximum flow rates, to attain the required content in the putamen [122]. However, this method resulted in tissue damage, as evidenced by MRI. Nevertheless, the lower flow rates were not good enough to induce a therapeutic response [123]. The other findings revealed that the flow back of the molecule inside the catheter prompted the dispensation of BDNF into the cerebrospinal fluid; therefore, it caused apoptotic cell death. This technique requires augmentation, such as implementing an intravenous mechanism that could cease the molecule from outflow and needle, which can consistently dispense BDNF within the preferred cerebral region [124]. The technique for delivering the gene is considered safe and effective for delivering BDNF to the targeted tissues while decreasing the growth of additional tissues [125]. This technique, which introduces the BDNF gene into the brain’s nuclear basal using an adenovirus as a viral vector, was studied in treating AD [126]. Other techniques use substances that could stimulate BDNF production and assimilation [20] *via* amplifying TrkB receptor mobilization through resistors like 7,8-DHF, a distinct approach has been put forward [127].

## CONCLUSION

One of the NTFs which regulate their role using the TrkB receptor is BDNF. Through perpetuating normal neuron conditions, BDNF performs a vital function in the CNS, which is undoubtedly expressed during memory and cognitive functions. The elderly phase and neurological diseases are linked to the BDNF gene’s diminished expression.

In this article, firstly, considering the prospect of being employed in neurotrophic rehabilitation for memory and cognitive functions, we have reviewed the role of BMP2 as a cofactor for BDNF. It will be essential to ascertain whether this BMP ligand effectively secures dopaminergic neurons. Secondly, the physiological consequence of autophagy in neuronal function is firmly surging. The studies herein divulge that autophagy is distinctively regulated in discrete brain sections. The increasing body of literature suggests that enhanced autophagy regulates the synaptic abnormalities carried by BDNF. Hence, in this review, we attempted to focus on the role of BDNF in autophagy, the main culprit behind cognitive impairment. Thirdly, through this review, we also attempted to discuss cognitive evaluation and behavior in neurological disorders, leading to improvements in early diagnosis and treatment.

Finally, at the end of this article, we summarised the role of BDNF as a protective agent against various neurological disorders, including schizophrenia, AD, PD, and HD. However, there is an utmost need for further studies to examine BDNF's function as a biomarker for disorders and diagnosis. We intend that this article will impart a basis for future research by providing more mechanistic intuition into neurological abnormalities emerging from the deregulation of neurotrophic signaling of BDNF, which are crucial for progressing target therapies.

## Figures and Tables

**Fig. (1):**
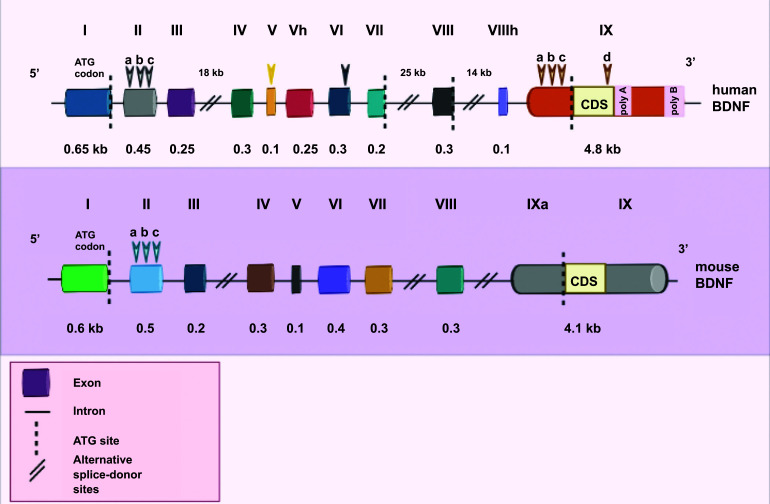
Comparison of structural organization of human, rat, and mouse BDNF. The human BDNF exhibits a high degree of similarity to the structural organization of rat and mouse BDNF.

**Fig. (3):**
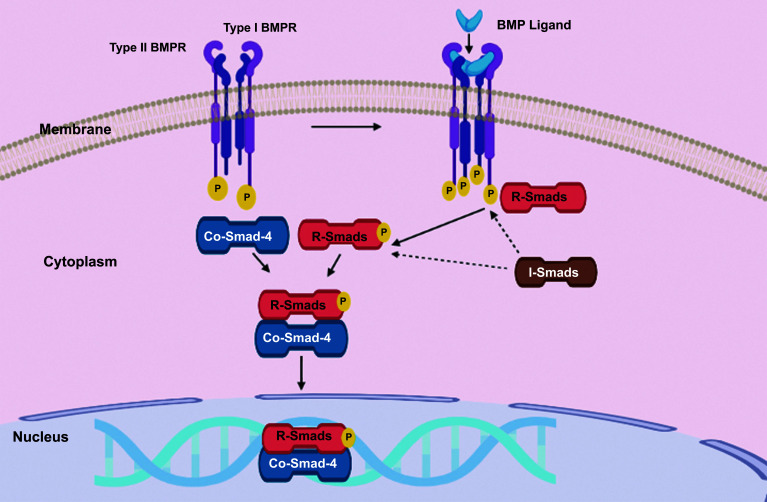
Phosphorylation of type I receptors by type II receptors upon ligand binding to the heterotetrameric receptor complex, as illustrated in Figure 3. The binding of the ligand triggers the phosphorylation of serine and threonine residues on the type I receptors by the type II receptors.

## LIST OF ABBREVIATIONS

7,8 DHF: 7,8-Dihydroxyflavone
AD: Alzheimer’s Disease
ALK: Activin Receptor-like Kinase
ALS: Amyotrophic Lateral Sclerosis
APP: Amyloid Precursor Protein
Aβ: Amyloid Peptides
BBB: Blood Brain Barrier
BDNF: Brain-derived Neurotrophic Factor
BMP: Bone Morphogenetic Protein
CBP: CREB-binding Protein
CI: Cognitive Impairment
CREB: cAMP Response Element-Binding Protein
DAG: Diacylglycerol
DOR: Dorsal Root Ganglion
ER: Endoplasmic Reticulum
ERK: Extracellular Signal-regulated Kinase
GDNF: Glial Cell-derived Neurotrophic Factor
GRB2: Growth Factor Receptor-bound Protein 2
GSK3: Glycogen Synthase Kinase 3
HD: Huntington’s Disease
IP3: Inositol Triphosphate
IRS-1: Insulin Receptor Substrate 1
ISH: Insulin Hybridisation
LC3: Microtubule-associated Protein Light Chain 3
LNGFR: Low-affinity Nerve Growth Factor Receptor
LTD: Long-term Depression
LTP: Long-Term Potentiation
MS: Multiple Sclerosis
mTOR: Mechanistic Target of Rapamycin
NFT: Neurofibrillary Tangles
NGF: Nerve Growth Factor
NMDA: N-methyl-D-aspartate
NP: Neuritic Plaques
NRF2: Nuclear Factor Erythroid 2-related Factor 2
NRTN: Neurturin
NT: Neurotrophin
NTF: Neurotrophic Factors
OB: Olfactory Bulb
PD: Parkinson’s Disease
PDK1: 3-Phosphoinositide-dependent Kinase1
PI3K: Phosphoinositide 3-Kinase
PKA: Protein Kinase A
PLC: Phospholipase C
SN: Substantia Nigra
SQSTM 1: Sequestosome 1
T2DM: Type 2 Diabetes Mellitus
TGF-β: Transforming Growth Factor-β
TRKB: Tropomyosin Receptor Kinase B

## References

[1] Binder D.K., Scharfman H.E. (2004). Brain-derived neurotrophic factor.. Growth Factors.

[2] Bathina S., Das U.N. (2015). Brain-derived neurotrophic factor and its clinical implications.. Arch. Med. Sci..

[3] Huang E.J., Reichardt L.F. (2001). Neurotrophins: Roles in neuronal development and function.. Annu. Rev. Neurosci..

[4] Berlanga-Macías C., Sánchez-López M., Solera-Martínez M. (2021). Relationship between exclusive breastfeeding and brain-derived neurotrophic factor in children.. PLoS One.

[5] Tanila H. (2017). The role of BDNF in Alzheimer's disease.Neurobiol Dis.

[6] Hong C.J., Liou Y.J., Tsai S.J. (2011). Effects of BDNF polymorphisms on brain function and behavior in health and disease.. Brain Res. Bull..

[7] Benraiss A., Chmielnicki E., Lerner K., Roh D., Goldman S.A. (2001). Adenoviral brain-derived neurotrophic factor induces both neostriatal and olfactory neuronal recruitment from endogenous progenitor cells in the adult forebrain.. J. Neurosci..

[8] Padmakumar S., Jones G., Pawar G. (2021). Minimally invasive nasal depot (MIND) technique for direct BDNF AntagoNAT delivery to the brain.. J. Control. Release.

[9] Shekari A. (2021). The effects of agind and alzheimer’s disease on retrograde neurotropin transport in basal forebrain cholinergic neurons..

[10] Scalzo P., Kümmer A., Bretas T.L., Cardoso F., Teixeira A.L. (2010). Serum levels of brain-derived neurotrophic factor correlate with motor impairment in Parkinson’s disease.. J. Neurol..

[11] Mughal M.R., Baharani A., Chigurupati S. (2011). Electroconvulsive shock ameliorates disease processes and extends survival in huntingtin mutant mice.. Hum. Mol. Genet..

[12] Sohrabji F., Lewis D.K. (2006). Estrogen–BDNF interactions: Implications for neurodegenerative diseases.. Front. Neuroendocrinol..

[13] Mo Y., Yao H., Lv W. (2016). Effects of electroacupuncture at governor vessel acupoints on neurotrophin-3 in rats with experimental spinal cord injury.. Neural Plast..

[14] Aid T., Kazantseva A., Piirsoo M., Palm K., Timmusk T. (2007). Mouse and ratBDNF gene structure and expression revisited.. J. Neurosci. Res..

[15] Arévalo J.C., Deogracias R. (2023). Mechanisms controlling the expression and secretion of BDNF.. Biomolecules.

[16] Hernández-Echeagaray E. (2020). The role of the TrkB-T1 receptor in the neurotrophin-4/5 antagonism of brain-derived neurotrophic factor on corticostriatal synaptic transmission.. Neural Regen. Res..

[17] Meeker R., Williams K. (2014). Dynamic nature of the p75 neurotrophin receptor in response to injury and disease.. J. Neuroimmune Pharmacol..

[18] Reichardt L.F. (2006). Neurotrophin-regulated signalling pathways.. Philos. Trans. R. Soc. Lond. B Biol. Sci..

[19] Ibrahim A.M., Chauhan L., Bhardwaj A. (2022). Brain-derived neurotropic factor in neurodegenerative disorders.. Biomedicines.

[20] Cocco E., Scaltriti M., Drilon A. (2018). NTRK fusion-positive cancers and TRK inhibitor therapy.. Nat. Rev. Clin. Oncol..

[21] Huang E.J., Reichardt L.F. (2003). Trk receptors: roles in neuronal signal transduction.. Annu. Rev. Biochem..

[22] Pandit M., Behl T., Sachdeva M., Arora S. (2020). Role of brain derived neurotropic factor in obesity.. Obes. Med..

[23] Pandya C.D., Kutiyanawalla A., Pillai A. (2013). BDNF–TrkB signaling and neuroprotection in schizophrenia.. Asian J. Psychiatr..

[24] Xu F., Na L., Li Y., Chen L. (2020). Roles of the PI3K/AKT/mTOR signalling pathways in neurodegenerative diseases and tumours.. Cell Biosci..

[25] Li H., Xue X., Li L. (2020). Aluminum-induced synaptic plasticity impairment via PI3K-Akt-mTOR signaling pathway.. Neurotox. Res..

[26] Mutti V., Bono F., Tomasoni Z. (2022). Structural plasticity of dopaminergic neurons requires the activation of the D3R-nAChR heteromer and the PI3K-ERK1/2/Akt-induced expression of c-Fos and p70S6K signaling pathway.. Mol. Neurobiol..

[27] Kolczynska K., Loza-Valdes A., Hawro I., Sumara G. (2020). Diacylglycerol-evoked activation of PKC and PKD isoforms in regulation of glucose and lipid metabolism: a review.. Lipids Health Dis..

[28] Zhao S., Shi J., Yu G. (2020). Photosensitive tyrosine analogues unravel site-dependent phosphorylation in TrkA initiated MAPK/ERK signaling.. Commun. Biol..

[29] Hisano K., Kawase S., Mimura T. (2021). Structurally different lysophosphatidylethanolamine species stimulate neurite outgrowth in cultured cortical neurons via distinct G-protein-coupled receptors and signaling cascades.. Biochem. Biophys. Res. Commun..

[30] Nasrolahi A., Javaherforooshzadeh F., Jafarzadeh-Gharehziaaddin M., Mahmoudi J., Asl K.D., Shabani Z. (2022). Therapeutic potential of neurotrophic factors in Alzheimer’s Disease.. Mol. Biol. Rep..

[31] Castelli V., Alfonsetti M., d’Angelo M. (2023). Neurotrophic factor-based pharmacological approaches in neurological disorders.. Neural Regen. Res..

[32] Sullivan A.M., O’Keeffe G.W. (2016). Neurotrophic factor therapy for Parkinson’s disease: past, present and future.. Neural Regen. Res..

[33] Voutilainen MH, Arumäe U, Airavaara M, Saarma M (2015). Therapeutic potential of the endoplasmic reticulum located and secreted CDNF/MANF family of neurotrophic factors in Parkinson’s disease. FEBS Lett.

[34] Barker R.A., Björklund A., Gash D.M. (2020). GDNF and Parkinson’s disease: where next? A summary from a recent workshop.. J. Parkinsons Dis..

[35] Wang J., Hu W.W., Jiang Z., Feng M.J. (2020). Advances in treatment of neurodegenerative diseases: Perspectives for combination of stem cells with neurotrophic factors.. World J. Stem Cells.

[36] Nordvall G., Forsell P., Sandin J. (2022). Neurotrophin-targeted therapeutics: A gateway to cognition and more?. Drug Discov. Today.

[37] Goulding S.R., Sullivan A.M., O’Keeffe G.W., Collins L.M. (2020). The potential of bone morphogenetic protein 2 as a neurotrophic factor for Parkinson’s disease.. Neural Regen. Res..

[38] Jann J., Gascon S., Roux S., Faucheux N. (2020). Influence of the TGF-β superfamily on osteoclasts/osteoblasts balance in physiological and pathological bone conditions.. Int. J. Mol. Sci..

[39] Sampath T.K., Reddi A.H. (2020). Discovery of bone morphogenetic proteins – A historical perspective.. Bone.

[40] Haraguchi R., Kitazawa R., Kohara Y., Ikedo A., Imai Y., Kitazawa S. (2019). Recent insights into long bone development: Central role of hedgehog signaling pathway in regulating growth plate.. Int. J. Mol. Sci..

[41] Sanchez-Duffhues G., Williams E., Goumans M.J., Heldin C.H., ten Dijke P. (2020). Bone morphogenetic protein receptors: Structure, function and targeting by selective small molecule kinase inhibitors.. Bone.

[42] Weiss A., Attisano L. (2013). The TGFbeta superfamily signaling pathway.. Wiley Interdiscip. Rev. Dev. Biol..

[43] Hanke T., Wong J.F., Berger B.T. (2020). A highly selective chemical probe for activin receptor-like kinases ALK4 and ALK5.. ACS Chem. Biol..

[44] Zhang Y.E. (2009). Non-Smad pathways in TGF-β signaling.. Cell Res..

[45] Hill C.S. (2009). Nucleocytoplasmic shuttling of Smad proteins.. Cell Res..

[46] Nickel J., Mueller T.D. (2019). Specification of BMP Signaling.. Cells.

[47] Miyazono K., Kamiya Y., Morikawa M. (2010). Bone morphogenetic protein receptors and signal transduction.. J. Biochem..

[48] Fu L., Cui C.P., Zhang X., Zhang L. (2020). The functions and regulation of Smurfs in cancers.. Semin. Cancer Biol..

[49] Goulding S.R. (2021). Defining the potential of gene therapy with bone morphogenetic proteins as a novel therapeutic approach in parkinson’s disease..

[50] Mueller T.D., Nickel J. (2012). Promiscuity and specificity in BMP receptor activation.. FEBS Lett..

[51] Renault L., Patiño L.C., Magnin F. (2020). BMPR1A and BMPR1B missense mutations cause primary ovarian insufficiency.. J. Clin. Endocrinol. Metab..

[52] Agnew C., Ayaz P., Kashima R. (2021). Structural basis for ALK2/BMPR2 receptor complex signaling through kinase domain oligomerization.. Nat. Commun..

[53] Schliermann A., Nickel J. (2018). Unraveling the connection between fibroblast growth factor and bone morphogenetic protein signaling.. Int. J. Mol. Sci..

[54] Hegarty S.V., Sullivan A.M., O’Keeffe G.W. (2017). Endocytosis contributes to BMP2-induced Smad signalling and neuronal growth.. Neurosci. Lett..

[55] Marincola Smith P., Means A., Beauchamp R. (2019). Immunomodulatory effects of TGF-β family signaling within intestinal epithelial cells and carcinomas.. Gastrointestinal Disorders.

[56] Anantha J., Goulding S.R., Wyatt S.L. (2020). STRAP and NME1 mediate the neurite growth-promoting effects of the neurotrophic factor GDF5.. iScience.

[57] Hegarty S.V., O’Keeffe G.W., Sullivan A.M. (2014). Neurotrophic factors: From neurodevelopmental regulators to novel therapies for Parkinson’s disease.. Neural Regen. Res..

[58] Goulding S.R., Sullivan A.M., O’Keeffe G.W., Collins L.M. (2019). Gene co-expression analysis of the human substantia nigra identifies BMP2 as a neurotrophic factor that can promote neurite growth in cells overexpressing wild-type or A53T α-synuclein.. Parkinsonism Relat. Disord..

[59] Nikoletopoulou V., Sidiropoulou K., Kallergi E., Dalezios Y., Tavernarakis N. (2017). Modulation of autophagy by BDNF underlies synaptic plasticity.. Cell Metab..

[60] Chen S.D., Wu C.L., Hwang W.C., Yang D.I. (2017). More insight into BDNF against neurodegeneration: anti-apoptosis, anti-oxidation, and suppression of autophagy.. Int. J. Mol. Sci..

[61] Zhu Z., Yang C., Iyaswamy A. (2019). Balancing mTOR signaling and autophagy in the treatment of Parkinson’s disease.. Int. J. Mol. Sci..

[62] Pringle E.S., Robinson C.A., McCormick C. (2019). Kaposi’s sarcoma-associated herpesvirus lytic replication interferes with mTORC1 regulation of autophagy and viral protein synthesis.. J. Virol..

[63] Yin Z., Popelka H., Lei Y., Yang Y., Klionsky D.J. (2020). The roles of ubiquitin in mediating autophagy.. Cells.

[64] Wu C.L., Chen C.H., Hwang C.S., Chen S.D., Hwang W.C., Yang D.I. (2017). Roles of p62 in BDNF-dependent autophagy suppression and neuroprotection against mitochondrial dysfunction in rat cortical neurons.. J. Neurochem..

[65] Colardo M., Martella N., Pensabene D. (2021). Neurotrophins as key regulators of cell metabolism: Implications for cholesterol homeostasis.. Int. J. Mol. Sci..

[66] Chottekalapanda R.U., Kalik S., Gresack J. (2020). AP-1 controls the p11-dependent antidepressant response.. Mol. Psychiatry.

[67] Daniele S., Giacomelli C., Martini C. (2018). Brain ageing and neurodegenerative disease: The role of cellular waste management.. Biochem. Pharmacol..

[68] Du T.T., Zhu G., Chen Y. (2020). Anterior thalamic nucleus stimulation protects hippocampal neurons by activating autophagy in epileptic monkeys.. Aging (Albany NY).

[69] Liu X., Jaenisch R. (2000). Severe peripheral sensory neuron loss and modest motor neuron reduction in mice with combined deficiency of brain-derived neurotrophic factor, neurotrophin 3 and neurotrophin 4/5.. Dev. Dyn..

[70] Zagrebelsky M., Tacke C., Korte M. (2020). BDNF signaling during the lifetime of dendritic spines.. Cell Tissue Res..

[71] Brunet A., Datta S.R., Greenberg M.E. (2001). Transcription-dependent and -independent control of neuronal survival by the PI3K–Akt signaling pathway.. Curr. Opin. Neurobiol..

[72] Klöcker N., Kermer P., Weishaupt J.H., Labes M., Ankerhold R., Bähr M. (2000). Brain-derived neurotrophic factor-mediated neuroprotection of adult rat retinal ganglion cells in vivo does not exclusively depend on phosphatidyl-inositol-3′-kinase/protein kinase B signaling.. J. Neurosci..

[73] Lee J., Seroogy K.B., Mattson M.P. (2002). Dietary restriction enhances neurotrophin expression and neurogenesis in the hippocampus of adult mice.. J. Neurochem..

[74] Rauti R., Cellot G., D’Andrea P. (2020). BDNF impact on synaptic dynamics: Extra or intracellular long-term release differently regulates cultured hippocampal synapses.. Mol. Brain.

[75] Colucci-D’Amato L., Speranza L., Volpicelli F. (2020). Neurotrophic factor BDNF, physiological functions and therapeutic potential in depression, neurodegeneration and brain cancer.. Int. J. Mol. Sci..

[76] Bathina S., Srinivas N., Das U.N. (2017). Streptozotocin produces oxidative stress, inflammation and decreases BDNF concentrations to induce apoptosis of RIN5F cells and type 2 diabetes mellitus in Wistar rats.. Biochem. Biophys. Res. Commun..

[77] Parveen R., Kapur P., Kohli S., Agarwal N.B. (2022). Attenuated brain derived neurotrophic factor and depression in type 2 diabetes mellitus patients: A case-control study.. Clin. Epidemiol. Glob. Health.

[78] Zhang X.Y., Liang J., Chen D.C. (2012). Low BDNF is associated with cognitive impairment in chronic patients with schizophrenia.. Psychopharmacology (Berl.).

[79] Wang Z.H., Xiang J., Liu X. (2019). Deficiency in BDNF/TrkB neurotrophic activity stimulates δ-secretase by upregulating C/EBPβ in alzheimer’s disease.. Cell Rep..

[80] Banerjee M., Shenoy R.R. (2023). Emphasizing roles of BDNF promoters and inducers in Alzheimer’s disease for improving impaired cognition and memory.. J. Basic Clin. Physiol. Pharmacol..

[81] Numakawa T., Odaka H. (2021). Brain-derived neurotrophic factor signaling in the pathophysiology of Alzheimer’s disease: Beneficial effects of flavonoids for neuroprotection.. Int. J. Mol. Sci..

[82] Ginsberg S.D., Malek-Ahmadi M.H., Alldred M.J. (2019). Brain-derived neurotrophic factor (BDNF) and TrkB hippocampal gene expression are putative predictors of neuritic plaque and neurofibrillary tangle pathology.. Neurobiol. Dis..

[83] Zuccato C., Cattaneo E. (2009). Brain-derived neurotrophic factor in neurodegenerative diseases.. Nat. Rev. Neurol..

[84] Rigamonti D., Sipione S., Goffredo D., Zuccato C., Fossale E., Cattaneo E. (2001). Huntingtin’s neuroprotective activity occurs via inhibition of procaspase-9 processing.. J. Biol. Chem..

[85] Zuccato C., Cattaneo E. (2007). Role of brain-derived neurotrophic factor in Huntington’s disease.. Prog. Neurobiol..

[86] Zuccato C., Ciammola A., Rigamonti D. (2001). Loss of huntingtin-mediated BDNF gene transcription in Huntington’s disease.. Science.

[87] Balaratnasingam S., Janca A. (2012). Brain Derived Neurotrophic Factor: A novel neurotrophin involved in psychiatric and neurological disorders.. Pharmacol. Ther..

[88] Nagahara A.H., Tuszynski M.H. (2011). Potential therapeutic uses of BDNF in neurological and psychiatric disorders.. Nat. Rev. Drug Discov..

[89] Sarchielli E., Marini M., Ambrosini S. (2014). Multifaceted roles of BDNF and FGF2 in human striatal primordium development. An in vitro study.. Exp. Neurol..

[90] Simmons D.A., Rex C.S., Palmer L. (2009). Up-regulating BDNF with an ampakine rescues synaptic plasticity and memory in Huntington’s disease knockin mice.. Proc. Natl. Acad. Sci. USA.

[91] Elkouzi A., Vedam-Mai V., Eisinger R.S., Okun M.S. (2019). Emerging therapies in Parkinson disease — repurposed drugs and new approaches.. Nat. Rev. Neurol..

[92] Sonne J., Reddy V., Beato M.R. (2022). Neuroanatomy, Substantia Nigra. StatPearls..

[93] André V.M., Cepeda C., Levine M.S. (2010). Dopamine and glutamate in Huntington’s disease: A balancing act.. CNS Neurosci. Ther..

[94] Palasz E., Wysocka A., Gasiorowska A., Chalimoniuk M., Niewiadomski W., Niewiadomska G. (2020). BDNF as a promising therapeutic agent in Parkinson’s disease.. Int. J. Mol. Sci..

[95] Bruna B., Lobos P., Herrera-Molina R., Hidalgo C., Paula-Lima A., Adasme T. (2018). The signaling pathways underlying BDNF-induced Nrf2 hippocampal nuclear translocation involve ROS, RyR-Mediated Ca2+ signals, ERK and PI3K.. Biochem. Biophys. Res. Commun..

[96] Singh K., Han K., Tilve S., Wu K., Geller H.M., Sack M.N. (2018). Parkin targets NOD2 to regulate astrocyte endoplasmic reticulum stress and inflammation.. Glia.

[97] Wang L., Li J., Di L. (2022). Glycogen synthesis and beyond, a comprehensive review of GSK3 as a key regulator of metabolic pathways and a therapeutic target for treating metabolic diseases.. Med. Res. Rev..

[98] Kang S.S., Zhang Z., Liu X. (2017). TrkB neurotrophic activities are blocked by α-synuclein, triggering dopaminergic cell death in Parkinson’s disease.. Proc. Natl. Acad. Sci. USA.

[99] Jin W. (2020). Regulation of BDNF-TrkB signaling and potential therapeutic strategies for Parkinson’s disease.. J. Clin. Med..

[100] Ni Y., Yang X., Zheng L. (2019). Lactobacillus and Bifidobacterium improves physiological function and cognitive ability in aged mice by the regulation of gut microbiota.. Mol. Nutr. Food Res..

[101] Julienne H., Buhl E., Leslie D.S., Hodge J.J.L. (2017). Drosophila PINK1 and parkin loss-of-function mutants display a range of non-motor Parkinson’s disease phenotypes.. Neurobiol. Dis..

[102] Tomassoni-Ardori F., Fulgenzi G., Becker J. (2019). Rbfox1 up-regulation impairs BDNF-dependent hippocampal LTP by dysregulating TrkB isoform expression levels.. eLife.

[103] Jourdi H., Hsu Y.T., Zhou M., Qin Q., Bi X., Baudry M. (2009). Positive AMPA receptor modulation rapidly stimulates BDNF release and increases dendritic mRNA translation.. J. Neurosci..

[104] Hernandez C.M., Terry A.V. (2005). Repeated nicotine exposure in rats: Effects on memory function, cholinergic markers and nerve growth factor.. Neuroscience.

[105] Mercado N.M., Collier T.J., Sortwell C.E., Steece-Collier K. (2017). BDNF in the aged brain: translational implications for Parkinson’s disease.. Austin Neurol. Neurosci..

[106] Vauzour D., Camprubi-Robles M., Miquel-Kergoat S. (2017). Nutrition for the ageing brain: Towards evidence for an optimal diet.. Ageing Res. Rev..

[107] Sahu G., Malavade K., Jacob T. (2016). Cognitive impairment in schizophrenia: Interplay of BDNF and childhood trauma? A review of literature.. Psychiatr. Q..

[108] Wang R., Holsinger R.M.D. (2018). Exercise-induced brain-derived neurotrophic factor expression: Therapeutic implications for Alzheimer’s dementia.. Ageing Res. Rev..

[109] Hashimoto K. (2014). Serum brain-derived neurotrophic factor as a predictor of incident dementia.. JAMA Neurol..

[110] Hashimoto K. (2013). Sigma-1 receptor chaperone and brain-derived neurotrophic factor: Emerging links between cardiovascular disease and depression.. Prog. Neurobiol..

[111] Dorszewska J. (2013). Cell biology of normal brain aging: Synaptic plasticity–cell death.. Aging Clin. Exp. Res..

[112] Galle S., Licher S., Milders M. (2021). Plasma brain-derived neurotropic factor levels are associated with aging and smoking but not with future dementia in the rotterdam study.. J. Alzheimers Dis..

[113] Enette L., Vogel T., Fanon J.L., Lang P.O. (2017). Effect of interval and continuous aerobic training on basal serum and plasma brain-derived neurotrophic factor values in seniors: A systematic review of intervention studies.. Rejuvenation Res..

[114] Wang C.S., Kavalali E.T., Monteggia L.M. (2022). BDNF signaling in context: From synaptic regulation to psychiatric disorders.. Cell.

[115] Fujisawa M., Takeshita Y., Fujikawa S. (2022). Exploring lipophilic compounds that induce BDNF secretion in astrocytes beyond the BBB using a new multi-cultured human in vitro BBB model.. J. Neuroimmunol..

[116] Chen C., Dong Y., Liu F. (2020). A study of antidepressant effect and mechanism on intranasal delivery of BDNF-HA2TAT/AAV to rats with post-stroke depression.. Neuropsychiatr. Dis. Treat..

[117] Pardridge W.M. (2020). Blood-brain barrier and delivery of protein and gene therapeutics to brain.. Front. Aging Neurosci..

[118] D’Souza A., Dave K.M., Stetler R.A.S., Manickam D. (2021). Targeting the blood-brain barrier for the delivery of stroke therapies.. Adv. Drug Deliv. Rev..

[119] Padmakumar S., Taha M.S., Kadakia E., Bleier B.S., Amiji M.M. (2020). Delivery of neurotrophic factors in the treatment of age-related chronic neurodegenerative diseases.. Expert Opin. Drug Deliv..

[120] Gravesteijn E., Mensink R.P., Plat J. (2022). Effects of nutritional interventions on BDNF concentrations in humans: A systematic review.. Nutr. Neurosci..

[121] Mohammadi A., Amooeian V.G., Rashidi E. (2018). Dysfunction in brain-derived neurotrophic factor signaling pathway and susceptibility to schizophrenia, Parkinson’s and Alzheimer’s diseases.. Curr. Gene Ther..

[122] Allen S.J., Watson J.J., Shoemark D.K., Barua N.U., Patel N.K. (2013). GDNF, NGF and BDNF as therapeutic options for neurodegeneration.. Pharmacol. Ther..

[123] Lang A.E., Gill S., Patel N.K. (2006). Randomized controlled trial of intraputamenal glial cell line-derived neurotrophic factor infusion in Parkinson disease.. Ann. Neurol..

[124] Hovland D.N., Boyd R.B., Butt M.T. (2007). Six-month continuous intraputamenal infusion toxicity study of recombinant methionyl human glial cell line-derived neurotrophic factor (r-metHuGDNF in rhesus monkeys.. Toxicol. Pathol..

[125] Leake P.A., Akil O., Lang H. (2020). Neurotrophin gene therapy to promote survival of spiral ganglion neurons after deafness.. Hear. Res..

[126] Lim S.T., Airavaara M., Harvey B.K. (2010). Viral vectors for neurotrophic factor delivery: A gene therapy approach for neurodegenerative diseases of the CNS.. Pharmacol. Res..

[127] Jang S.W., Liu X., Yepes M. (2010). A selective TrkB agonist with potent neurotrophic activities by 7,8-dihydroxyflavone.. Proc. Natl. Acad. Sci. USA.

